# Disinfection and Disinfectants

**Published:** 1885-06

**Authors:** 


### Disinfection and Disinfectants.

[We publish this month the most important parts of the paper issued by the American Public Health Association. We feel that this paper is just what is wanted at this season, and it is the best treatise we have seen on the subject. We regret that our space prevents us from printing the entire article.—Ed.]

The object of *disinfection* is to prevent the extension of infectious diseases by destroying the specific infectious material which gives rise to them. This is accomplished by the use of *disinfectants*.

There can be no partial disinfection of such material; either its infecting power is destroyed or it is not. In the latter case there is a failure to disinfect Nor can there be any disinfection in the absence of infectious material.

It has been proved for several kinds of infectious material that its specific infecting power is due to the presence of living micro-organisms, known in a general way as "disease germs;" and practical sanitation is now based upon the belief that the infecting agents in all kinds of infectious material are of this nature. Disinfection, therefore consists essentially in the destruction of disease germs.

Popularly, the term disinfection is used in a much broader sense. Any chemical agent which destroys or masks bad odors, or which arrests putrefactive decomposition is spoken of as a disinfectant. And in the absence of any infectious disease it is common to speak of disinfecting a foul cess-pool, or bad-smelling stable, or privy vault.

This popular use of the term has led to much misapprehension, and the agents which have been found to destroy bad odors—*deodorisers*—or to arrest putrefactive decomposition—*antiseptics*—have been confidently recommended and extensively used for the destruction of disease germs in the excreta of patients with cholera, typhoid fever, etc.

The injurious consequences which are likely to result from such misapprehension and misuse of the word disinfectant will be appreciated when it is known that:

*Recent researches have demonstrated that many of the agents which have been found useful as deodorisers, or as antiseptics, are entirely without value for the destruction of disease germs.*

This is true, for example, as regards the sulphate of iron or copperas, a salt which has been extensively used with the idea that it is a valuable disinfectant. As a matter of fact, sulphate of iron in saturated solution does not destroy the vitality of disease germs or the infecting power of material containing them. This salt is, nevertheless, a very valuable antiseptic, and its low price makes it one of the most available agents for the arrest of putrefactive decomposition in privy vaults, etc.

*Antiseptic agents also exercise a restraining influence upon the development of disease germs, and their use during epidemics is to be recommended, when masses of organic material in the vicinity of human habitations can not be completely destroyed, or removed, or disinfected.*

While an antiseptic agent is not necessarily a disinfectant, all disinfectants are antiseptics ; for putrefactive decomposition is due to the development of "germs" of the same class as that to which disease germs belong, and the agents which destroy the latter also destroy the bacteria of putrefaction, when brought in contact with them in sufficient quantity, or restrain their development when present in smaller amounts.

*A large number of the proprietary "disinfectants," so called, which are in the market, are simply deodorisers or antiseptics, of greater or less value, and are entirely untrustworthy for disinfecting purposes.*

Antiseptics are to be used at all times when it is impracticable to remove filth from the vicinity of human habitations, but they are a poor substitute for cleanliness.

Dnring the prevalence of epidemic diseases, such as yellow fever, typhoid fever and cholera, it is better to use, in privy-vaults, cesspools, etc., those antiseptics which are also disinfectants—i. e., germicides ; and when the contents of such receptacles are known to be infected this becomes imperative.

*In the sick-room we have disease germs at an advantage, for we know where to find them as well as how to kill them.*

Having this knowledge, not to apply it would be criminal negligence, for our efforts to restrict the extension of infectious diseases must depend largely upon the proper use of disinfectants in the sick-room.

GENERAL DIRECTIONS.

*Disinfection of Excreta, etc.*—The infectious character of the ejections of patients suffering from cholera and typhoid fever is well established ; and this is true of mild cases and of the earliest stages of these diseases as well as of severe and fatal cases. It is probable that epidemic dysentery, tuberculosis, and perhaps diphtheria, yellow fever, scarlet fever and typhus fever may also be transmitted by means of the alvine discharges of the sick. It is therefore of the first importance that these should be disinfected. In cholera, diphtheria, yellow fever and scarlet fever, all vomited material should also be looked upon as infectious. And in tuberculosis, diphtheria, scarlet fever and infectious pneumonia, the sputa of the sick should be disinfected or destroyed by fire. It seems advisable also to treat the urine of patients, sick with an infectious disease, with one of the disinfecting solutions below recommended.

*Chloride of lime,* or bleaching powder, is, perhaps, entitled to the first place for disinfecting excreta, on account of the rapidity of its action. The following standard solution is recommended :

*Standard Solution No.* 1.—Dissolve chloride of lime of the best quality in soft water, in the proportion of four ounces to the gallon.

Use one pint of this solution for the disinfection of each discharge in cholera, typhoid fever, etc. Mix well and leave in vessel for at least ten minutes before throwing into privy-vault or water-closet. The same directions apply for the disinfection of vomited matters. Infected sputum should be discharged directly into a cup half full of the solution.

*Standard Solution No.* 2.—Dissolve Corrosive Sublimate and Permanganate of Potash in soft water, in the proportion of two drachms of each salt to the gallon.

This is to be used for the same purposes and in the same way as Standard Solution No. 1. It is equally effective, but it is necessary to leave it for a longer time in contact with the material to be disinfected—at least an hour.

[Great care must be taken in using or preparing any mixture of corrosive sublimate. It is one of the most active poisons. For this reason Solution No. 1 should always be used where possible instead of the above.—Ed.]

*Standard Solution No.* 3.—To one part of Labarraque's Solution (liquor sodæ chlorinatæ), add five parts of soft water.

It may be used in the same manner as recommended for Standard Solution No. 1.

### DISINFECTING AND ANTISEPTIC POWDERS.

*One pound of chloride of lime; one ounce of corrosive sublimate; nine pounds of plaster of Paris. Pulverize the corrosive sublimate and mix thoroughly with the plaster of Paris. Then add the chloride of lime and mix well. Pack in paste-board boxes or in wooden casks. Keep dry.*

As an antiseptic and deodorizer this powder is to be sprinkled upon the surface of excreta, etc.

To disinfect excreta in the sick room, cover the entire surface with a thin layer of the powder—one-fourth inch in thickness—and if the material is not liquid pour on sufficient water to cover it.

*Disinfection of the Person.*—The surface of the body of a sick person, or of his attendants, when soiled with infectious discharges, should be at once cleansed with a suitable disinfecting agent. For this purpose Standard Solution No. 3 may be used.

In diseases like small-pox and scarlet fever, in which the infectious agent is given off from the entire surface of the body, occasional ablutions with Labarraque's Solution, diluted with twenty parts of water, will be more suitable than the stronger solution above recommended.

In all infectious diseases the surface of the body of the dead should be thoroughly washed with one of the standard solutions above recommended, and then enveloped in a sheet saturated with the same.

*Disinfection of Clothing.*—Boiling for half an hour will destroy the vitality of all known disease germs, and there is no better way of disinfecting clothing or bedding which can be washed than to put it through the ordinary operations of the laundry. No delay should occur, however, between the time of removing soiled clothing from the person or bed of the sick and its immersion in boiling water, or in some disinfectant solution and no article should be permitted to leave the infected room until so treated.

Clothing may also be disinfected by immersion for two hours in a solution made by diluting Standard Solution No. 1 with nine parts of water—one gallon in ten.

*Disinfection of the sick room.*—In the sick room no disinfectant can take the place of free ventilation and cleanliness. It is an axiom in sanitary science that *it is impracticable to disinfect an occupied apartment;* for the reason that disease germs are not destroyed by the presence in the atmosphere of any known disinfectant in respirable quantity Bad odors may be neutralized, but this does not constitute disinfection in the sense in which the term is here used. These bad odors are, for the most part, an indication of want of cleanliness, or of proper ventilation ; and it is better to turn contaminated air out of the window, or up the chimney, than to attempt to puri y it by the use of volatile chemical agents, such as carbolic acid, chlorine, etc., which are all more or less offensive to the sick, and are useless so far as disinfection—properly so-called—is concerned.

*When an apartment which has been occupied by a person sick with an infectious disease is vacated, it should be disinfected.* But it is hardly worth while to attempt to disinfect the atmosphere of such an apartment, for this will escape through an open window and be replaced by fresh air from without, while preparations are being made to disinfect it. Moreover, experience shows that the infecting power of such an atmosphere is quickly lost by dilution, or by the destruction of floating disease germs through contact with oxygen, and that even small-pox and scarlet fever are not transmitted to any great distance through the atmosphere; while cholera, typhoid fever and yellow fever are rarely, if ever, contracted by contact with the sick, or by respiring the atmosphere of the apartments occupied by them.

The object of disinfection in the sick room is mainly the destruction of infectious material attached to surfaces, or deposited as dust upon window-ledges, in crevices, etc. If the room has been properly cleansed and ventilated while still occupied by the sick person, and especially if it was stripped of carpets and unnecessary furniture at the outset of his attack, the difficulties of disinfection will be greatly reduced.

All surfaces should be thoroughly washed with a solution of corrosive sublimate of the strength of one part in 1000 parts of water. The walls and ceiling, if plastered, should be brushed over with this solution, after which they may be whitewashed with a lime wash. Especial care must be taken to wash away all dust from window-ledges and other places where it may have settled, and to thoroughly cleanse crevices and out-of-the-way places. After this application of the disinfecting solution, and an interval of twenty-four hours or longer for free ventilation, the floors and wood-work should be well scrubbed with soap and hot water, and this should be followed by a second and more prolonged exposure to fresh air, admitted through open doors and windows.

*Disinfection of ingesta.*—It is well established that cholera and typhoid fever are very frequently and, perhaps, usually transmitted through the medium of infected water or articles of food, and especially milk. Fortunately we have a simple means at hand for disinfecting such infected fluids. This consists in the application of heat. *The boiling temperature maintained for half an hour kills all known disease germs.* So far as the germs of cholera, yellow fever and diphtheria are concerned, there is good reason to believe that a temperature considerably below the boiling point of water will destroy them. But in order to keep on the safe side it is best not to trust anything short of the boiling point (212 deg. F.) when the object in view is to disinfect food or drink which is open to the suspicion of containing the germs of any infectious disease.

During the prevalence of an epidemic of cholera it is well to boil all water for drinking purposes. After boiling, the water may be filtered, if necessary to remove sediment, and then cooled with *pure* ice if desired.

A sheet of filtering paper, such as druggists use, and a glass or tin funnel, furnishes the best means for filtering water on a small scale for drinking purposes. A fresh sheet of paper is to be used each day.

